# Ocular pharmacokinetics of tivozanib eye drops in patients with neovascular age-related macular degeneration: comparison with phase 1 trials of oral tivozanib in solid tumors

**DOI:** 10.1186/s40942-026-00848-9

**Published:** 2026-04-15

**Authors:** Shinya Horita, Mayumi Saki, Sotaro Takigawa, Yasumasa Kanai, Kenta Murotani, Fumi Gomi

**Affiliations:** 1 Development Division, Kyowa Kirin Co., Ltd., Tokyo, Japan; 2 Medical Affairs Division, Kyowa Kirin Co., Ltd., Tokyo, Japan; 3 School of Medical Technology, Kurume University, Kurume-shi, Fukuoka Japan; 4 Biostatistics Center, Kurume University, Kurume-shi, Fukuoka Japan; 5 Department of Ophthalmology, Hyogo Medical University, Nishinomiya-shi, Hyogo Japan

**Keywords:** Choroid, Hypertension, Pharmacokinetics, Receptors, vascular endothelial growth factor, Retina, Tivozanib, Drug delivery, Eye drops, Neovascular age-related macular degeneration, Posterior eye tissues

## Abstract

Intravitreal anti-vascular endothelial growth factor (VEGF) therapy is the standard treatment for neovascular age-related macular degeneration (nAMD) but is invasive and burdensome for patients. To overcome these obstacles, tivozanib, a pan–VEGF receptor inhibitor approved for renal cell carcinoma, is in development as eye drops. The phase 1 study of a 3-week administration of tivozanib eye drops showed a preferable safety profile and exploratory efficacy signs in Japanese patients with nAMD. During eye drop administration, two drug delivery pathways affect ocular pharmacokinetics, either directly through the ocular tissues or through systemic circulation. To assess the ocular pharmacokinetics of tivozanib eye drops, we conducted an integrated analysis of systemic adverse event occurrence and systemic exposure in patients with nAMD treated with tivozanib eye drops and in patients with cancer treated with oral tivozanib, using previous phase 1 study results. Systemic exposure was significantly lower with tivozanib eye drops versus oral tivozanib, with no drug-related hypertension due to the systemic pan–VEGF receptor inhibition observed, suggesting systemic VEGF receptors are not fully inhibited by tivozanib. Exploratory efficacy signs in patients with nAMD after short-term use of tivozanib eye drops also suggest tivozanib might reach the retina and inhibit VEGF receptors, although further long-term efficacy studies are warranted. Given the integrated analysis, we hypothesize that tivozanib eye drops could predominantly be delivered to the posterior eye segment through the ocular tissues, with limited contribution by the systemic drug delivery pathway. This hypothesis provides meaningful insights into the ocular pharmacokinetics of tivozanib eye drops.

## Background

Age-related macular degeneration (AMD) is a chronic, progressive, degenerative disease that affects the macular region of the retina [1]. Neovascular AMD (nAMD), which accounts for 15–20% of all AMD cases [2], is characterized by choroidal neovascularization (CNV), vascular leakage, hemorrhage, and retinal exudation, and it can result in blindness [2, 3]. Vascular endothelial growth factor (VEGF) and its receptors are major mediators involved in the pathophysiology of nAMD [4]. Intravitreal anti-VEGF therapies have shown efficacy and safety in patients with nAMD [5–8] and are the current standard-of-care treatment for nAMD [4]. However, the need for frequent and repeated administration, along with regular clinic visits, and fear and anxiety related to invasive intravitreal injections can present patients with substantial treatment and economic burdens [9–12]. Moreover, intravitreal anti-VEGF therapies may increase the risk of endophthalmitis [13] and be associated with other ocular complications, such as conjunctival hemorrhage and floaters [14, 15]. Therefore, it is increasingly recognized that eye drops may potentially serve as an alternative non-invasive treatment option for nAMD to overcome or mitigate the challenges associated with intravitreal injections.

### Drug delivery pathways in the administration of eye drops to patients with nAMD

In general, topical instillation of eye drops involves two potential pathways to deliver the drug to the target posterior ocular tissues (e.g. choroid, retina) [16, 17]: the local ocular pathway where the drug directly penetrates through the anterior ocular tissues to reach the posterior eye segments [16], and the systemic pathway where the drug initially enters the systemic circulation, subsequently distributing to the posterior ocular tissues [17]. When the local ocular pathway is predominant, the active ingredient of the eye drops effectively reaches the posterior eye tissues with minimal systemic drug exposure and systemic adverse events (AEs). On the other hand, when the systemic pathway is predominant, achieving therapeutic drug concentrations in the posterior ocular tissues requires elevated systemic drug exposure, potentially increasing the likelihood of systemic AEs. In the case of administration of anti-VEGF/VEGF receptor drugs, elevated systemic drug exposure can lead to systemic VEGF/VEGF receptor inhibition and may cause VEGF/VEGF receptor inhibition–related AEs such as hypertension [18, 19]. Therefore, ideally, eye drops with a small-molecule VEGF receptor inhibitor as an active ingredient intended for the treatment of nAMD should work predominantly via the local ocular pathway for high local drug exposure, specifically in the posterior ocular tissues, to minimize systemic drug exposure. This allows sufficient local inhibition of the VEGF receptor pathway and therapeutic efficacy while minimizing systemic drug exposure and the risks of systemic VEGF receptor–related AEs. Therefore, in the clinical development of eye drops for nAMD treatment, assessment and understanding of the drug delivery pathways (local ocular vs. systemic pathways) are important to characterize the drug’s pharmacokinetic (PK)/pharmacological profile, and to ensure a balance between efficacy and safety.

However, in the administration of eye drops, achieving sufficient drug exposure in the posterior ocular tissues predominantly via the local ocular pathway can be challenging. Indeed, regorafenib and pazopanib eye drops, which were developed for nAMD treatment, demonstrated lack of efficacy in their phase 2 trials [20, 21]. This is possibly due to low bioavailability of these compounds after topical instillation as eye drops that led to insufficient drug exposure in the posterior ocular tissues. For this reason, further development of these eye drops was terminated. A PK study of regorafenib and pazopanib eye drops in rats, rabbits, and monkeys suggested notable interspecies differences in ocular anatomy, structure, and physiological functions that may affect the drug’s ocular PK and efficacy [22]. Despite both regorafenib and pazopanib eye drops demonstrating favorable posterior ocular PK and efficacy profiles in rats, this was not observed in larger animals such as rabbits and monkeys, where substantially lower drug concentrations were reported in the posterior ocular tissues following topical administration of the eye drops. In rats, the concentrations of regorafenib were substantially higher in the dosed eye versus the non-dosed eye, suggesting the contribution of the local ocular pathway. In contrast, low regorafenib concentration was detected in both the dosed and non-dosed eyes of rabbits and monkeys, suggesting neither local nor systemic pathways provided sufficient drug delivery to the posterior ocular tissues. Similarly, for pazopanib eye drops, a substantially higher drug concentration was observed in the dosed eye versus the non-dosed eye in rats. In monkeys, however, its concentration was comparable between the dosed and non-dosed eyes, suggesting a dominant contribution of the local ocular pathway in rats but of the systemic pathway in monkeys.

Tivozanib is a potent and selective small-molecule pan–VEGF receptor tyrosine kinase inhibitor originally developed as an oral formulation for the treatment of advanced renal cell carcinoma (RCC) [23]. Oral tivozanib demonstrated clinical efficacy and a favorable safety profile in phase 3 clinical trials of RCC [24, 25] and has been approved for advanced RCC treatment in Europe and the United States. While generally well tolerated, tivozanib is associated with AEs related to systemic inhibition of VEGF receptors, including hypertension [26]. Specifically, hypertension occurred in 46.8% of patients, with 20.2% of patients experiencing a grade ≥ 3 event. Tivozanib is currently in development as eye drops for the treatment of nAMD and diabetic macular edema by applying nano-crystallization technology to enhance drug delivery to the posterior eye tissues. The phase 1 study of tivozanib eye drops in healthy volunteers and Japanese patients with nAMD reported a favorable safety profile and exploratory efficacy signs [27]. In this phase 1 study, most AEs and drug-related AEs were ocular events; importantly, no drug-related hypertension was observed. Mean (standard deviation [SD]) central subfield thickness (CST) decreased from baseline to Day 22 by − 27.6 (54.88), − 35.6 (49.64), and − 43.7 (55.19) µm in tivozanib doses of 0.45 mg/day (0.5% weight/volume [w/v], one drop three times a day [TID]), 0.9 mg/day (1.0% w/v, one drop TID), and 1.8 mg/day (1.0% w/v, two drops TID), respectively [27]. Tivozanib eye drops also maintained or improved best-corrected visual acuity (BCVA) score in 24 of 28 patients, suggesting positive clinical signs that warrant further investigation [27]. Moreover, these findings imply the involvement of the local ocular pathway in drug delivery, with tivozanib reaching the posterior ocular tissues and inhibiting the VEGF receptor pathway locally with minimal systemic exposure. However, to date, the ocular PK and drug delivery pathway of tivozanib eye drops have not been fully elucidated.

### Assessment of the ocular PK profile of eye drops targeting nAMD

One of the major challenges in assessing ocular PK in clinical practice is the inability to directly measure drug concentrations in the target posterior ocular tissues. While aqueous humor and the vitreous body may be collected in clinical studies, sampling of the retina and choroids is typically not feasible from clinical and ethical perspectives. Moreover, the complex structure and composition of the retina and choroid, including various proteins and melanin pigments, may also hamper the precise determination of the tissue drug composition and the unbound drug fraction relevant to the pharmacological activity of the drug in the ocular tissues. Additionally, evaluation of the pharmacological effects in the posterior eye tissues is also not sufficient to fully characterize the ocular PK of eye drops because of the presence of both local ocular and systemic pathways. For example, patients with nAMD with subfoveal CNV treated with an intravenous infusion of bevacizumab, an anti-VEGF antibody, had improved visual acuity (VA) in the study eyes within the first 2 weeks of treatment [28]. In these patients, mean VA letter scores increased by 14 letters in the study eye (*p* < 0.001), and mean central retinal thickness significantly decreased by 112 μm (*p* < 0.001), by 24 weeks. However, these patients also had elevated systolic and diastolic blood pressure by 3 weeks. These results suggest that, while bevacizumab infusion works through the systemic pathway, its concentration reached an efficacious level to inhibit the VEGF pathway in the ocular tissues also. Despite its efficacy in the eye, the intravenous infusion of bevacizumab was not considered to have a reasonable benefit–risk profile as a treatment for nAMD due to its systemic AEs. Therefore, to better understand the ocular PK and the contributions of the local ocular and systemic pathways in the drug delivery of tivozanib eye drops, we proposed a new approach to integrate clinical efficacy with systemic drug exposure and the occurrence of systemic AEs, specifically hypertension.

### Evidence-based assessment of contributions of the local ocular and systemic pathways on the ocular PK profile of tivozanib eye drops

We assessed the serum drug concentration and the incidence of hypertension as a drug-related event—the most typical and sensitive AE caused by the inhibition of the VEGF receptor pathway—with tivozanib eye drops versus oral tivozanib, by comparing clinical data from a phase 1 study of nAMD [27] with pooled clinical data from two phase 1 studies of solid tumors [29, 30]. In the phase 1 studies of solid tumors, patients in Japan were treated with oral tivozanib at doses of 1.0 and 1.5 mg/day, and patients in the Netherlands were treated with oral tivozanib at doses of 1.0, 1.5, and 2.0 mg/day. When data from these two studies were pooled, mean (SD) values of maximum concentration (C_max_) at steady state (Japan: Cycle 1 Days 21–22; the Netherlands: Cycle 1 Day 28) at doses of 1.0, 1.5, and 2.0 mg/day were 49.75 (20.91), 76.23 (46.20), and 110.00 (61.43) ng/mL, respectively, with high rates of hypertension reported in a dose-dependent manner in these patients (1.0 mg/day: 38.1%; 1.5 mg/day: 59.1%; and 2.0 mg/day: 100.0%). These results were consistent with the most robust phase 2 and phase 3 data of oral tivozanib [24, 25, 31]. Conversely, in Japanese patients with nAMD treated with tivozanib eye drops, mean (SD) concentration at steady state (Day 22) at doses of 0.45 mg/day (0.5% w/v, one drop TID), 0.9 mg/day (1.0% w/v, one drop TID), and 1.8 mg/day (1.0% w/v, two drops TID) was 5.84 (2.12), 16.8 (7.4), and 29.2 (22.9) ng/mL, respectively, with healthy volunteers demonstrating a comparable PK profile to those with nAMD [27]. Across all dose groups, no hypertension as a drug-related AE was reported in these patients during the study period.

**Fig. 1:**
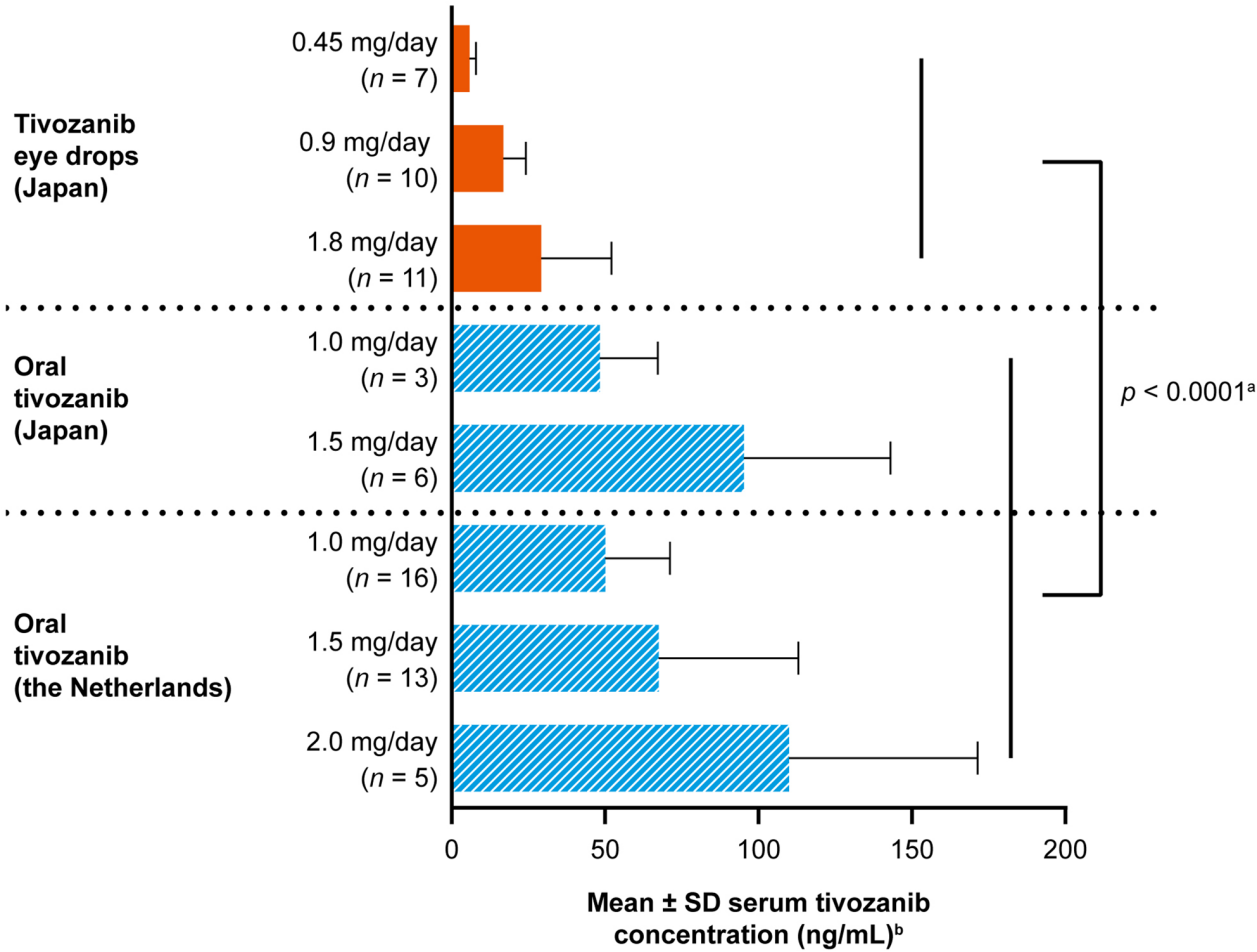
Mean ± SD serum tivozanib concentration. Note: Doses of tivozanib eye drops were 0.45 mg/day (0.5% w/v, one drop TID), 0.9 mg/day (1.0% w/v, one drop TID), and 1.8 mg/day (1.0% w/v, two drops TID). Serum tivozanib concentration was measured in Japanese patients with nAMD on Day 22 of treatment with tivozanib eye drops [27], and in patients with solid tumors in Japan and in the Netherlands on Cycle 1 Days 21–22 and Cycle 1 Day 28, respectively, of treatment with oral tivozanib [29, 30]. ^a^*p*-value was calculated using Welch’s t-test for serum tivozanib concentration with eye drops (Japan [*N* = 28]) vs oral (Japan and the Netherlands [*N* = 43]). ^b^Serum tivozanib concentration was the serum concentration at steady state for tivozanib eye drops and C_max_ at steady state for oral tivozanib. Abbreviations: C_max_, maximum concentration; nAMD, neovascular age-related macular degeneration; SD, standard deviation; TID, three times a day; w/v, weight/volume

Our analysis revealed that serum drug concentration at steady state in patients with nAMD treated with tivozanib eye drops (*N* = 28) was significantly lower compared with the C_max_ of the drug in patients with solid tumors treated with oral tivozanib (*N* = 43) (mean [SD] 18.93 [15.18] vs. 68.46 [39.34] ng/mL, Welch’s t-test, *p* < 0.0001; Fig. 1). The results were consistent with the comparison of Japanese patients only (mean [SD] 18.93 [15.18] vs. 79.58 [41.59] ng/mL, Welch’s t-test, *p* = 0.0022) [27, 29]. Additionally, the incidence of drug-related hypertension was significantly lower with tivozanib eye drops than with oral tivozanib (0% [0/28] vs. 56.0% [28/50], Fisher’s exact test, *p* < 0.0001; Table 1); similar results were observed in the comparison of Japanese patients only (eye drops: 0% [0/28], oral: 44.4% [4/9], Fisher’s exact test, *p* = 0.0019) [27, 29].

**Table 1:** Incidence of tivozanib-related hypertension

Route of Administration	Country	Dose, mg/day	*n*	Grade 1/2	Grade 3/4	All Grades	Overall	*p*-value^a^
Eye drops	Japan [27]^b^	0.45	7	0 (0)	0 (0)	0 (0)	0 (0)	< 0.0001
		0.9	10	0 (0)	0 (0)	0 (0)		
		1.8	11	0 (0)	0 (0)	0 (0)		
Oral	Japan [29]^c^	1.0	3	1 (33.3)	0 (0)	1 (33.3)	28 (56.0)	
		1.5	6	1 (16.7)	2 (33.3)	3 (50.0)		
	The Netherlands [30]^d^	1.0	18	2 (11.1)	5 (27.8)	7 (38.9)		
		1.5	16	0 (0)	10 (62.5)	10 (62.5)		
		2.0	7	2 (28.6)	5 (71.4)	7 (100.0)		

Data are presented as *n* (%)

Note: Doses of tivozanib eye drops were 0.45 mg/day (0.5% w/v, one drop TID), 0.9 mg/day (1.0% w/v, one drop TID), and 1.8 mg/day (1.0% w/v, two drops TID)

^a^*p*-value was calculated using Fisher’s exact test for the overall incidence of hypertension occurring with eye drops (Japan [0/28; 0%]) versus oral (Japan and the Netherlands [28/50; 56.0%])

^b^Graded according to the National Cancer Institute Common Terminology Criteria for Adverse Events Version 5.0

^c^Graded according to the National Cancer Institute Common Terminology Criteria for Adverse Events Version 3.0

^d^Details of grading are unavailable

Abbreviations: TID, three times a day; w/v, weight/volume

From an efficacy perspective, Gomi et al. [27] reported a decrease in retinal thickness and improvements in retinal morphology with 3-week treatment of tivozanib eye drops in patients with nAMD. Mean (SD) CST with doses of 0.45, 0.9, and 1.8 mg/day was 412.9 (161.18), 410.7 (103.28), and 413.3 (230.01) µm, respectively, at baseline, and 385.3 (119.89), 375.1 (92.25), and 369.5 (195.47) µm, respectively, at Day 22; CST decreased from baseline to Day 22 with all three doses, with a mean (SD) change from baseline of − 27.6 (54.88), − 35.6 (49.64), and − 43.7 (55.19) µm, respectively. Substantial anatomical changes, assessed by optical coherence tomography, were observed in 12 patients; intraretinal fluid disappeared in one patient on Day 22, and subretinal fluid disappeared in one patient on Day 8 and in four patients on Day 22. Additionally, the rate of dry macula was 14.3% on Day 22. These results suggest that eye drops may be able to deliver the active ingredient, tivozanib, to the posterior ocular tissues to exert local inhibition of the VEGF receptor pathway.

### Limitations

Our analyses were based on aggregated published data without multivariate adjustments because we were unable to obtain individual patient data from the clinical trials included in the analyses. Of note, the mean age of patients in Japan with nAMD was 71 years [27], the mean age of patients in the Netherlands with solid tumors was 56 years [30], and the median age of patients in Japan with solid tumors was 54 years [29]. Moreover, because data on the C_max_ of tivozanib eye drops were not available, we could not directly compare the C_max_ of tivozanib eye drops with the C_max_ of oral tivozanib. However, due to the long half-life of tivozanib eye drops (~ 100 h) [27], intraday fluctuations at steady state would be minimal, suggesting that the serum tivozanib concentration at steady state (Day 22) may be considered nearly equivalent to the C_max_ of the tivozanib eye drops. In fact, there was only a small difference between the C_max_ and the serum drug concentration at steady state (Day 22) in healthy participants receiving tivozanib eye drops [27].

### Consideration

From the phase 1 exploratory efficacy results of tivozanib eye drops [27], and this current analysis showing lower serum drug concentration and no incidence of hypertension with tivozanib eye drops versus oral tivozanib, we hypothesize that tivozanib is predominantly delivered to the posterior ocular tissues via the local ocular pathway following topical instillation of the eye drops. With minimal involvement of the systemic pathway, tivozanib eye drops can possibly maintain low systemic drug exposure and prevent systemic VEGF receptor inhibition–related AEs while ensuring local inhibition of the VEGF receptor pathway in the posterior ocular tissues. Our new hypothesis on tivozanib’s ocular PK and drug delivery pathway provides meaningful insights into the development of tivozanib eye drops, particularly when evaluating the benefit–risk profile, determining the optimal dose setting, and ultimately predicting the success of the treatment.

## Data Availability

All data generated or analyzed during this study are included in this published article.

## Abbreviations

AE: Adverse event
AMD: Age-related macular degeneration
C_max_: Maximum concentration
CNV: Choroidal neovascularization
CST: Central subfield thickness
nAMD: Neovascular AMD
PK: Pharmacokinetics
RCC: Renal cell carcinoma
SD: Standard deviation
TID: Three times a day
VA: Visual acuity
VEGF: Vascular endothelial growth factor
w/v: Weight/volume
